# Impaired insulin-stimulated myocardial glucose metabolic rate is associated with reduced estimated myocardial energetic efficiency in subjects with different degrees of glucose tolerance

**DOI:** 10.1186/s12933-022-01733-z

**Published:** 2023-01-09

**Authors:** Elena Succurro, Francesco Cicone, Annalisa Papa, Sofia Miceli, Patrizia Vizza, Teresa Vanessa Fiorentino, Maria Perticone, Angela Sciacqua, Pietro Hiram Guzzi, Pierangelo Veltri, Giuseppe Lucio Cascini, Francesco Andreozzi, Giorgio Sesti

**Affiliations:** 1 Department of Medical and Surgical Sciences, University Magna Graecia of Catanzaro, Viale Europa, 88100 Catanzaro, Italy; 2 Research Center for the Prevention and Treatment of Metabolic Diseases (CR METDIS), University Magna Graecia of Catanzaro, Catanzaro, Italy; 3 Department of Experimental and Clinical Medicine, Magna Graecia University of Catanzaro, Catanzaro, Italy; 4 Department of Clinical and Molecular Medicine, University of Rome-Sapienza, 00189 Rome, Italy

**Keywords:** Myocardial glucose metabolism, Myocardial mechano-energetic efficiency, Cardiovascular disease, Insulin sensitivity, Type 2 diabetes, Prediabetes, Cardiac ^18^F-FDG PET, Cardiac workload

## Abstract

**Background:**

Alterations in myocardial mechano-energetic efficiency (MEEi), which represents the capability of the left ventricles to convert the chemical energy obtained by oxidative metabolism into mechanical work, have been associated with cardiovascular disease. Although whole-body insulin resistance has been related to impaired myocardial MEEi, it is unknown the relationship between cardiac insulin resistance and MEEi. Aim of this study was to evaluate the relationship between insulin-stimulated myocardial glucose metabolic rate (MrGlu) and myocardial MEEi in subjects having different degrees of glucose tolerance.

**Methods:**

We evaluated insulin-stimulated myocardial MrGlu using cardiac dynamic positron emission tomography (PET) with ^18^F-Fluorodeoxyglucose (^18^F-FDG) combined with euglycemic-hyperinsulinemic clamp, and myocardial MEEi in 57 individuals without history of coronary heart disease having different degrees of glucose tolerance. The subjects were stratified into tertiles according to their myocardial MrGlu values.

**Results:**

After adjusting for age, gender and BMI, subjects in I tertile showed a decrease in myocardial MEEi (0.31 ± 0.05 vs 0.42 ± 0.14 ml/s*g, P = 0.02), and an increase in myocardial oxygen consumption (MVO_2_) (10,153 ± 1375 vs 7816 ± 1229 mmHg*bpm, P < 0.0001) as compared with subjects in III tertile. Univariate correlations showed that insulin-stimulated myocardial MrGlu was positively correlated with MEEi and whole-body glucose disposal, and negatively correlated with waist circumference, fasting plasma glucose, HbA1c and MVO_2_. In a multivariate regression analysis running a model including several CV risk factors, the only variable that remained significantly associated with MEEi was myocardial MrGlu (β 0.346; P = 0.01).

**Conclusions:**

These data suggest that an impairment in insulin-stimulated myocardial glucose metabolism is an independent contributor of depressed myocardial MEEi in subjects without history of CHD.

## Background

Type 2 diabetes (T2DM) and prediabetes are metabolic disorders associated with increased cardiovascular morbidity and mortality compared to general non-diabetic population [1–8]. Compelling evidence suggests that insulin resistance play a key role in the pathogenesis of T2DM [9, 10], and predicts the development of cardiovascular disease (CVD) also in non-diabetic subjects [11, 12]. Furthermore, a reduced whole-body insulin sensitivity has been shown to be an independent prognostic cardiovascular risk factor in patients with heart failure [13].

An impaired insulin-stimulated myocardial glucose metabolism is an alteration strongly correlated with whole-body insulin resistance and observed in patients with T2DM with or without coronary heart disease (CHD) or heart failure and in conditions at increased risk of T2DM, including prediabetes and metabolic syndrome [14–19]. Additionally, it has been shown that an impairment in myocardial glucose uptake is associated with coronary atherosclerosis and predicts CV events in patients with CHD [20, 21].

A compromised myocardial mechano-energetic efficiency (MEE) has recently emerged as an independent predictor of both CV events and heart failure [22, 23]. The myocardial MEE represents the capability of the left ventricle to convert the chemical energy obtained by oxidative metabolism into mechanical work. Left ventricular (LV) work can be represented by the force needed to eject blood (stroke volume, SV) into the aorta, and estimated as stroke work (SW). Energy to support cardiac work is provided almost exclusively by aerobic oxidation of substrate, with close coupling between myocardial oxygen consumption (MVO_2_) and LV structure and function [24]. Under normal conditions, the proportion of produced energy that is used for contraction is approximately 25%, and the residual energy is mainly dissipated as heat [25]. LV efficiency may be defined as the ratio between external work delivered by cardiomyocytes (i.e. SW) and the amount of energy produced for each contraction [26]. At a given external work, increased energetic expenditure results in lower values of MEE. Thus, low MEE might contribute to progression of overt cardiovascular disease [27, 28]. DeSimone et al. have validated a simple non-invasive method for estimating myocardial MEE based on determination of SW and MVO_2_ normalized for LV mass (MEEi) which provides the estimate of the ideal amount of blood pumped by each gram of LV mass in 1 s [23, 29]. A reduced myocardial MEEi has been observed in individuals with obesity, prediabetes, T2DM, non-alcoholic fatty liver disease (NAFLD) [23, 30–34]. Although whole-body insulin resistance has been related to an impairment of myocardial MEEi [31, 33, 34], to date has not yet been explored the link between cardiac insulin resistance and MEEi.

In the attempt to address this issue, we aimed to evaluate the relationship between insulin-stimulated myocardial glucose metabolic rate (MrGlu) using dynamic myocardial positron emission tomography (PET) with ^18^F-Fluorodeoxyglucose (^18^F-FDG) combined with euglycemic-hyperinsulinemic clamp, and myocardial MEEi in individuals without history of CHD having different degrees of glucose tolerance.

## Methods

### Study participants

The study cohort comprised 57 subjects participating in the CATAnzaro MEtabolic RIsk factors (CATAMERI), an ongoing observational study recruiting adult individuals with one or more cardio-metabolic risk factors recruited at a referral hospital of the University “Magna Graecia” of Catanzaro [15, 35]. Eligible subjects were recruited according to the following inclusion criteria: age between 30 and 70 years, and positivity for one or more cardio-metabolic risk factors including family history of diabetes, dysglycemia, hypertension, dyslipidemia, and overweight/obesity. Exclusion criteria were type 1 diabetes, end-stage renal disease, previous cardiovascular disease on the basis of medical history, resting electrocardiogram and stress test or myocardial scintigraphy for individuals with T2DM, history of atrial fibrillation or other arrhythmias, right and left bundle branch block, dyssynchrony in ventricular contraction, valvular heart disease, liver cirrhosis, history of malignant or autoimmune diseases, acute or chronic infections, history of alcohol or drug abuse and treatment with drugs known to influence glucose tolerance such as steroids and estro-progestins and medicaments affecting heart function including beta blockers and antiarrhythmic drugs. All subjects underwent anthropometrical evaluation including measurements of body mass index (BMI), waist circumference and body composition by bioelectrical impedance. Readings of blood pressure (BP) were obtained in the left arm of the supine patients, after 5 min of rest, using a standard sphygmomanometer. BP values were the average of three measurements after a 10 min period of rest in the supine position. After an overnight fasting, biochemical determinations and a 75 g OGTT was performed in individuals with FPG < 126 mg/dl, HbA1c < 6.5% and no history of T2DM. According to the ADA criteria [36], individuals were classified as having normal glucose tolerance (NGT) when fasting plasma glucose was < 100 mg/dl (5.5 mmol/l), 2-h postload glucose < 140 mg/dl (< 7.77 mmol/l) and HbA1c < 5.7%, prediabetes when fasting plasma glucose was 100–125 mg/dl (5.5–6.9 mmol/l), 2-h postload glucose 140–199 mg/dl (7.77–11.0 mmol/l) or HbA1c 5.7–6.4%, T2DM when fasting plasma glucose was ≥ 126 mg/dl (> 7 mmol/l), 2-h post-load glucose was ≥ 200 mg/dl (> 11.1 mmol/l), HbA1c ≥ 6.5% or in treatment with antidiabetic drugs.

On the second day, after 12-h fasting, all subjects underwent first echocardiography, and, then, ^18^F-FDG PET scan combined with euglycemic hyperinsulinemic clamp in the morning.

The study was approved by the Ethical Committee (Comitato Etico Azienda Ospedaliera “Mater Domini”), and informed consent was obtained from each subject in accordance with principles of the Declaration of Helsinki.

### ^*18*^*F-FDG PET scan combined with euglycemic hyperinsulinemic clamp*

Myocardial glucose metabolic rate (MrGlu) was measured by ^18^F-FDG-PET acquired during an euglycemic hyperinsulinemic clamp as previously described [19, 37]. Subjects received a priming dose of insulin (100 UI/mL) (Humulin R; Eli Lilly) during the initial 10 min to raise the serum insulin concentration acutely (80 mU/m2 × min), and then it was maintained by continuous insulin infusion fixed at 40 mU/m2 × min [38]. The blood glucose level was maintained constant at 90 mg/dl for the next 120 min by infusing 20% glucose at varying rates according to blood glucose measurements performed at 5-min intervals (mean coefficient of variation of blood glucose was < 4%). Glucose metabolized by the whole body (M) was calculated as the mean rate of glucose infusion measured during the last 60 min of the clamp examination (steady state) and was expressed as milligrams per minute per kilogram fat-free mass (M_FFM_).

The ^18^F-FDG-PET imaging procedure was performed on a hybrid PET/CT scanner (GE Discovery ST8- 2D PET scanner), starting 60 min after the insulin infusion. A 60-min dynamic acquisition was started simultaneously with the intravenous injection of 370 MBq^18^F-FDG, according to the following time frame sampling: 8 × 15 s, 2 × 30 s, 2 × 120 s, 1 × 180 s, 6 × 300 s, 2 × 600 s [39]. PET images were reconstructed in a 128 × 128 matrix using a OSEM algorithm, and corrected for decay and attenuation based on co-registered CT. The insulin-glucose infusion continued during the entire PET acquisition. The estimation of myocardial MrGlu was performed by Patlak compartmental modelling [15, 40], using the graphical tool specific for cardiac images analysis (PCARD) implemented in PMOD Software platform (Version 3.806) [39]. In PCARD, the full dynamic study is used for MRGlu calculation, and the arterial input function is extracted from a volume of interest (VOI) semi-automatically placed in the left ventricular cavity [40].

### Echocardiographic measurements

Tracings were taken with participants in a partial left decubitus position using a VIVID-7 Pro ultrasound machine (GE Technologies, Milwaukee, WI, USA) with an annular phased array 2.5-MHz transducer. All the readings were performed by the same experienced investigator to optimize the reproducibility, blinded to the clinical data of the examined individuals. Tracings were recorded under two-dimensional guidance, and M-mode measurements were taken at the tip of the mitral valve or just below. LV end-diastolic (LVEDV) and end-systolic volume (LVESV) were measured according to Simpson method and indexed for body surface area (BSA) [41]. Measurements of interventricular septum thickness (IVS), posterior wall thickness (PWT) were made at end-diastole. LV mass (LVM) was calculated using the Devereux formula [42] and normalized by BSA [LVMI]) [41, 43].

### Myocardial mechano-energetic efficiency measurements

The myocardial mechano-energetic efficiency (MEE) can be defined as the ratio between the external systolic work, and the amount of total energy produced for each contraction [21, 22, 29, 31]. External myocardial work can be estimated as stroke work (SW), with SW being computed as systolic blood pressure (SBP) x echocardiographic stroke volume (SV). SV was calculated as the difference between LV end-diastolic and end-systolic volumes by the z-derived method [29, 31].

Myocardial oxygen consumption (MVO_2_) reflects the total amount of energy produced by the myocardium, and can be estimated using the “double product” (DP) of SBP x heart rate (HR) [31]. Thus, MEE may be estimated as: SBP × SV/ SBP × HR = SV/HR where HR were expressed in seconds (HR/60). Because MEE is highly related to LV mass, MEE was normalized for LV mass with the purpose of obtaining an estimate of energetic expenditure per unit of myocardial mass (i.e. indexed MEE, MEEi, ml/s/g) [21, 22, 29, 31].

### Laboratory determinations

Plasma glucose, total and HDL cholesterol, and triglycerides were assayed using enzymatic methods (Roche Diagnostics, Mannheim, Germany). HbA1c was measured with high performance liquid chromatography using an NGSP-certified automated analyzer (Adams HA-8160 HbA1c analyzer, Menarini, Italy).

### Statistical analyses

Variables with skewed distribution, such as triglycerides and M_FFM_, were natural log transformed for statistical analyses. Continuous variables are expressed as means ± SD. Categorical variables were compared by χ^2^ test. Comparisons between study groups were performed using a general linear model with post hoc Fisher's least significant difference correction for pairwise comparisons. Relationships between variables were determined by Pearson’s correlation coefficient (r). Stepwise multivariate regression analysis was run to determine the independent contributors of myocardial MEEi.

Considering that previous studies have reported a reduction of 10–17% of myocardial MEEi in subjects with different degrees of glucose tolerance in whom insulin resistance was evaluated with HOMA-IR [31, 34], we calculated that 17 subjects for each group had 80% power to detect a 10% difference in myocardial MEEi with an alpha of 0.05. With an addition of 15% to safeguard from potential missing values, a sample size of 19 subjects for each group was planned.

For all analyses, a P value ≤ 0.05 was considered to be statistically significant. All analyses were performed using SPSS software Version 22 for Windows.

## Results

Of the 57 recruited subjects, 20 (35.1%) had NGT, 11 (19.3%) had prediabetes, and 26 (45.6%) had T2DM. Clinical characteristics of the three groups of subjects stratified into tertiles according to their insulin-stimulated myocardial MrGlu values are shown in Table 1. No differences were observed in sex distribution. Subjects in the tertile I of insulin-stimulated myocardial MrGlu (range myocardial MrGlu 0.1–16.3 μmol/min/100 g) were older and exhibited higher BMI than individuals in the tertile III (range myocardial MrGlu 26.3–43 μmol/min/100 g) (Table 1). All the subjects with type 2 diabetes were treated with metformin.

**Table 1:** Anthropometric and metabolic characteristics of study subjects stratified into tertiles according to myocardial MrGlu values

	Myocardial MrGlu Tertile 1 Range 0.1–16.3 μmol*/min/100 g* (1)	Myocardial MrGlu Tertile 2 Range 16.4–26.29 μmol*/min/100 g* (2)	Myocardial MrGlu Tertile 3 Range 26.3–43 μmol*/min/100 g* (3)	*P (*1 vs 2)§	*P (1* vs 3)§	*P (2* vs 3)§
Sex (F/M)	10/9	8/11	9/10	0.41	0.87	0.5
Age (years)	53 ± 10	50 ± 12	48 ± 11	0.9	0.08	0.09
BMI (Kg/m^2^)	32.5 ± 5	28.1 ± 4	29.2 ± 4	0.01	0.02	0.6
Waist circumference (cm)	108 ± 12	98 ± 11	101 ± 10	0.5	0.4	0.7
Current smokers (%)	47.4	52.9	31.6	0.8	0.09	0.2
Systolic blood pressure (mmHg)	130 ± 12	124 ± 16	115 ± 15	0.5	0.03	0.1
Diastolic blood pressure (mmHg)	79 ± 11	75 ± 11	75 ± 10	0.9	0.9	0.7
Heart Rate (bpm)	78 ± 8	68 ± 7	68 ± 4	< 0.0001	< 0.0001	0.4
Fasting Plasma Glucose (mg/dL)	131 ± 45	114 ± 35	100 ± 27	0.1	0.054	0.3
2-h post load plasma glucose (mg/dl)	149 ± 15	132 ± 24	115 ± 13	0.1	< 0.0001	0.07
HbA1c (%)	7.1 ± 1.2	6.5 ± 1.1	5.8 ± 1.1	0.2	0.01	0.1
Total Cholesterol (mg/dl)	186 ± 37	196 ± 47	185 ± 29	0.2	0.7	0.3
HDL Cholesterol (mg/dl)	45 ± 10	48 ± 8	49 ± 14	0.3	0.5	0.8
LDL Cholesterol (mg/dl)	126 ± 36	127 ± 36	118 ± 28	0.6	0.9	0.3
Triglycerides (mg/dl)	164 ± 74	119 ± 64	115 ± 60	0.2	0.1	0.7
NGT/Prediabetes/T2DM (n)	1/4/14	6/6/7	13/1/5	0.005	0.001	0.2
Insulin-stimulated glucose disposal (mg/min x Kg FFM)	3.16 ± 1.8	4.8 ± 3.1	8.4 ± 7.7	0.1	0.02	0.2
Antihypertensive therapy (%)	58.3	35.3	16.7	0.4	0.04	0.1
Glucose-lowering therapy (%) Meftormin (%)	70.6	33.3	26.3	0.02	0.01	0.8

Data are means ± SD, unless otherwise indicated. Categorical variables were compared by χ^2^ test. Comparisons between the three groups were performed using a general linear model with post hoc Fisher's least significant difference correction for pairwise comparisons.

^§^P values refer to results after analyses with adjustment for age, gender and BMI

*BMI* Body mass index, *NGT* normal glucose tolerance, *T2DM* type 2 diabetes

### Cardiovascular risk factors and metabolic parameters in subjects stratified according to insulin-stimulated myocardial MrGlu values

As shown in Table 1, no differences between the three groups were observed in waist circumference, lipid profile, fasting plasma glucose, diastolic blood pressure and proportion of current smokers (Table 1). After adjusting for age, sex and BMI, individuals in the tertile I showed higher values of systolic blood pressure, resting heart rate, 2-h post load plasma glucose and HbA1c than subjects in the tertile III (Table 1). Furthermore, a higher proportion of subjects in the tertile I had prediabetes or T2DM, and were treated with antihypertensive and glucose-lowering therapies than subjects in the tertile III (Table 1).

After adjusting for age, sex and BMI, individuals in the tertile I showed a significant reduction in the whole-body insulin-stimulated glucose disposal as compared with subjects in the tertile III (3.16 ± 1.8 vs 8.4 ± 7.7 mg/min x Kg FFM, P = 0.02) (Table 1). These differences remained significant after further adjustment for antidiabetic and antihypertensive therapy (P = 0.03) but not after adjustment for glucose tolerance status (P = 0.06).

A greater proportion of subjects in the tertile I had T2DM and were treated with glucose-lowering therapies as compared with subjects in the tertile II (Table 1). Furthermore, individuals in the tertile I exhibited higher values of resting heart rate as compared with subjects in the tertile II (Table 1). No further differences in cardiovascular and metabolic parameters were observed between individuals in the II and in the III myocardial MrGlu tertiles (Table 1).

### Myocardial mechano-energetic efficiency in subjects stratified according to insulin-stimulated myocardial MrGlu values

**Fig. 1:**
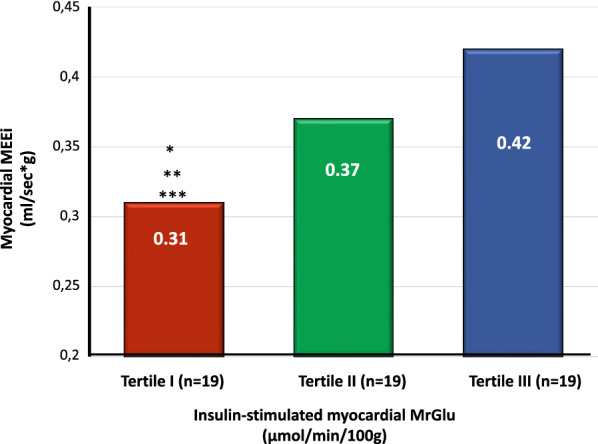
Myocardial MEEi (ml/sec*g-1) in subjects stratified into tertiles according to insulin-stimulated myocardial MrGlu (μmol/min/100 g). *P value refers to analyses after adjustment for age, gender and BMI *P* = 0.02 vs highest tertile. **After further adjustment for antidiabetic and antihypertensive therapy *P* = 0.03. ***After further adjustment for glucose tolerance status *P* = 0.04

Left ventricular geometry and energetic efficiency parameters of the study subjects stratified into tertiles according to insulin-stimulated myocardial MrGlu values are shown in Table 2. No differences between the three groups were observed in LV end-diastolic and end-systolic volumes, LVMI, interventricular septal thickness and posterior wall thickness. After adjusting for age, sex and BMI, subjects in the tertile I showed a significant decrease in myocardial MEEi as compared with individuals in the tertile III (0.31 ± 0.05 vs 0.42 ± 0.14 ml/sec*g, P = 0.02) (Table 2, Fig. 1). These differences remained significant after further adjustment for antidiabetic and antihypertensive therapy (P = 0.03) and for glucose tolerance status (P = 0.04). Furthermore, subjects in the tertile I exhibited a significant increase in myocardial workload estimated by MVO_2_ (10,153 ± 1375 mmHg*bpm) as compared with individuals in the tertile III (7816 ± 1229 mmHg*bpm, P < 0.0001) (Table 2). These differences remained significant after further adjustment for antidiabetic and antihypertensive therapy (P = 0.001) and for glucose tolerance status (P = 0.002). Moreover, subjects in the tertile I showed a significant increase in MVO_2_ as compared also individuals in the tertile II (8516 ± 1667 mmHg*bpm, P = 0.006) (Table 2). These differences remained significant after further adjustment for antidiabetic and antihypertensive therapy (P = 0.02) and for glucose tolerance status (P = 0.03).

**Table 2:** Left ventricular geometry and energetic efficiency parameters of the study subjects stratified into tertiles according to myocardial MrGlu values

	Myocardial MrGlu Tertile 1 Range 0.1–16.3 μmol*/min/100 g* (1)	Myocardial MrGlu Tertile 2 Range 16.4–26.29 μmol*/min/100 g* (2)	Myocardial MrGlu Tertile 3 Range 26.3–43 μmol*/min/100 g* (3)	*P (*1 vs 2)	*P (1* vs 3) §	*P (2* vs 3)§
LV end-systolic volume (LVESV) (*ml*)	38.3 ± 15	43.8 ± 11	40.9 ± 14	0.2	0.8	0.3
LV end-diastolic volume (LVEDV) (*ml*)	111 ± 31	121 ± 22	112 ± 23	0.2	0.6	0.5
Interventricular septal thickness (IVS) (*cm)*	1.13 ± 0.13	1.11 ± 0.12	1.05 ± 0.14	0.5	0.2	0.2
Posterior wall thickness (PWT) (*cm*)	0.87 ± 0.11	0.86 ± 0.12	0.84 ± 0.15	0.8	0.4	0.6
LV mass index (LVMI) (*g/m*^*2*^)	85.3 ± 22	93.1 ± 16	82.01 ± 18	0.1	0.6	0.07
Myocardial MEEi (ml/sec*g-1)	0.31 ± 0.05	0.37 ± 0.12	0.42 ± 0.14	0.1	0.02	0.4
Myocardial oxygen consumption (MVO2)(mmHg*bpm)	10,153 ± 1375	8516 ± 1667	7816 ± 1229	0.006	< 0.0001	0.1

Data are means ± SD, unless otherwise indicated. Comparisons between the three groups were performed using a general linear model with post hoc Fisher's least significant difference correction for pairwise comparisons

^§^P values refer to results after analyses with adjustment for age, gender and BMI

### Association between insulin-stimulated myocardial MrGlu, cardiovascular risk factors, and myocardial mechano-energetic efficiency

**Fig. 2:**
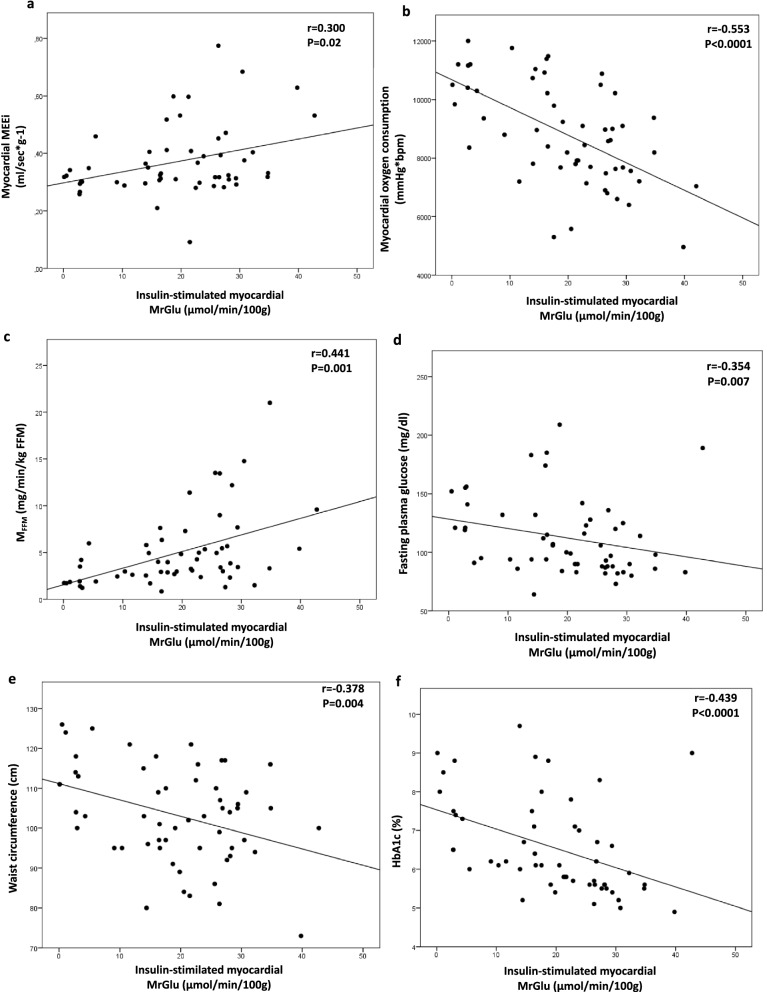
Relationship between insulin-stimulated myocardial MrGlu and myocardial MEEi (**a**), myocardial oxygen consumption (**b**), M_FFM_ (**c**), fasting plasma glucose (**d**), waist circumference (**e**), HbA1c (**f**)

**Fig. 3:**
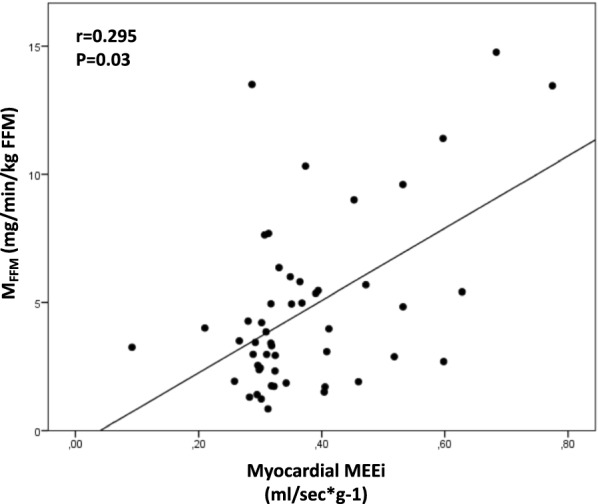
Relationship between myocardial MEEi and M_FFM_

Univariate correlations showed that insulin-stimulated myocardial MrGlu was negatively correlated with waist circumference (r = − 0.378, P = 0.004), fasting plasma glucose (r = -0.354, P = 0.007), HbA1c (r = − 0.439, P < 0.0001), and MVO_2_ (r = − 0.553, P < 0.0001) and positively correlated with myocardial MEEi (r = 0.300, P = 0.02), and whole-body insulin-stimulated glucose disposal (r = 0.441, P = 0.001) (Fig. 2). Furthermore, myocardial MEEi was positively correlated with whole-body insulin-stimulated glucose disposal (r = 0.295, P = 0.03) (Fig. 3). By contrast, no significant correlations between myocardial MEEi and fasting plasma glucose (r = − 0.08; P = 0.5) and HbA1c (r = − 0.16; P = 0.2) were detected.

To evaluate the independent contributors to myocardial MEEi, we performed a stepwise multivariate regression analysis running a model including age, gender, BMI, waist circumference, diastolic blood pressure, lipid profile, fasting plasma glucose, HbA1c, smoking, glucose tolerance status, antidiabetic therapy, insulin-stimulated glucose disposal and myocardial MrGlu. The only variable that remained significantly associated with myocardial MEEi was myocardial MrGlu (β 0.346; P = 0.01) explaining 34.6% of its variation.

## Discussion

The novel finding of this study was that cardiac insulin resistance estimated using dynamic ^18^F-FDG-PET combined with euglycemic-hyperinsulinemic clamp is an independent contributor of myocardial mechano-energetic efficiency, considered an emergent marker of CV risk [23]. Notably, we found that in subjects having different degrees of glucose tolerance without history of CHD impaired insulin-stimulated myocardial glucose metabolic rate was associated with a progressive decrease in myocardial MEEi paralleled by an increase in cardiac oxygen consumption, estimated using the “double product” of sBP x HR. Although cardiac energy and myocardial oxygen consumption can precisely be measured invasively by coronary sinus catheterization [26], these measurements are not feasible in routine clinic evaluation. A well-validated index of myocardial oxygen consumption is the “double product” which has been reported to have a high correlation with myocardial O_2_ consumption (r = 0.86–0.88) measured directly with coronary sinus catheterization [44, 45]. Similarly, cardiac energetic efficiency was estimated using myocardial MEEi, a simple, non-invasive, ultra-sound guided method, validated by DeSimone et al. that provides an estimate of the ideal amount of blood ejected at each systole per each gram of LV mass in 1 s [22, 23, 29, 31]. Reduced myocardial MEEi has been shown to be an independent predictor of major CV events in hypertensive patients [22]. Furthermore, low myocardial MEEi was reported to be a powerful predictor of heart failure in subjects with normal ejection fraction [23]. Additionally, a reduced myocardial MEEi has been shown in patients with primary aldosteronism, suggesting its role in determining their increased risk of CV events [46]. Moreover, low myocardial MEE is a predictor of mortality and poor prognosis also in patients with advanced chronic liver disease [47].

In our cross-sectional study, we show a direct relationship between cardiac insulin resistance and depressed myocardial energetic efficiency. Notably, in a stepwise multivariate linear regression analysis, after adjustment for several confounding factors, insulin-stimulated myocardial glucose metabolic rate was the only independent contributor of myocardial MEEi, explaining 34.6% of its variation. Our findings confirm and expand the knowledge on the role of insulin resistance in determining impairment of left ventricular efficiency [23, 30–34]. A decrease in myocardial MEEi has been observed in conditions of insulin resistance, including obesity, prediabetes, T2DM, and NAFLD [23, 30–34]. Furthermore, Mancusi et al. have demonstrated that severity of insulin resistance, assessed by the HOMA-IR index, had a negative impact on myocardial MEEi in nondiabetic individuals [31]. Noteworthy, in our report the myocardial MEEi values were lower than those reported in the study of Mancusi and coll. This disparity may be due to the different study population included (non-diabetic population vs subjects with different degrees of glucose tolerance, including T2DM in our study). Additionally, previous studies have confirmed an independent relationship between myocardial MEEi and insulin resistance, estimated by the Matsuda Index or the HOMA-IR index, in nondiabetic individuals [32, 33]. Of note, the present data represents the first demonstration of a direct independent correlation between myocardial MEEi and whole-body insulin sensitivity, assessed by the gold standard euglycemic-hyperinsulinemic clamp technique [38], in subjects having different degrees of glucose tolerance.

We also found a negative correlation between insulin-stimulated myocardial MrGlu and cardiac oxygen consumption in subjects without history of CHD. Overall, our findings support the idea that an impairment in insulin-stimulated myocardial glucose metabolism might contribute to the early compromission of left ventricular mechano-energetic efficiency, thus playing a significant role in the development of CVD. Our data extend previous findings [14] suggesting that cardiac insulin resistance may cause the development of CHD and heart failure in individuals with T2DM. An impairment in myocardial glucose uptake has been shown to be a predictor of adverse CV outcome also in subjects with ischemic heart disease [20]. Thus, a decrease in myocardial energetic performance could represent the pathogenetic mechanism through which cardiac insulin resistance contributes to the progression to CVD. Indeed, in a normal myocardium, 60–70% of energy is produced by free fatty acid (FFA) oxidation, while only 30–40% of energy is produced by glucose-pyruvate oxidation [31]. The ratio of produced ATP/MVO_2_ is higher with glucose (P/O = 2.58) than with FFA (P/O = 2.28), which produces a redundant number of ATP molecules, a source of energy that is mainly dissipated as heat [48, 49]. Accordingly, myocardial mechano-energetic efficiency is significantly higher when utilizing glucose rather than FFA. Under conditions of insulin resistance, the myocardium reduces glucose intake, leading to a substrate shift toward FFA oxidation. Consequently, the efficiency of cardiac energy utilization decreases, and the myocardium loses its metabolic flexibility. This reduction in cardiac efficiency coupled with raised oxygen demand increases susceptibility to myocardial ischemia leading to a greater reduction in myocardial performance [14, 18, 19, 21, 31, 48–54]. Clearly, further prospective studies are needed to confirm the causal role of the cardiac insulin resistance in the progression of CHD.

This study has some strengths that merit considerations. A main strength of the present study is the use of gold standard methods to assess myocardial and whole-body metabolism by cardiac FDG PET combined with the euglycemic-hyperinsulinemic clamp technique, which allows the valuation of insulin-stimulated myocardial glucose uptake under uniform experimental conditions of euglycemia and physiological hyperinsulinemia by removing the confounding factor of different circulating glucose and insulin levels [14, 55]. Moreover, glucose tolerance was accurately assessed using FPG, 2 h post-load glucose levels during an OGTT, and HbA1c according to ADA criteria thus excluding any potential misclassification of participants [36]. Additionally, all tests including echocardiographic measurements and ^18^F-FGD PET scan combined with euglycemic hyperinsulinemic clamp were collected by skilled examiners after a standardized training, who were blinded to the clinical data of the study participants.

Nonetheless, this study has also some limitations. Myocardial mechano-energetic efficiency was estimated by indirect measures rather than by coronary sinus catheterization [26]. However, this measurement is invasive, expensive, and time-consuming thus making this procedure not feasible in epidemiological studies. Nevertheless, adjustment of MEE for LVM and not for LVMI could have led to a lack of standardization between obese and non-obese subjects. Moreover, this analysis of the CATAMERI cohort study includes only Caucasian individuals aging between 30 and 70 years with at least one cardiovascular risk factors attending a referral university hospital, thus limiting the generalizability of the present results to other ethnicities or to white Caucasians cohorts. Furthermore, the overall sample size was small. Additionally, adjusting for several factors in a small population might produce misleading results [56, 57]. However, our planned sample size allows for adequate statistical power and going beyond this number would cause unnecessary radiation exposure to additional subjects. Additionally, the cross-sectional design and the observational nature of this study do not permit any causal inferences. Furthermore, we did not measure FFA levels, whose values are known to potentially influence the preferential substrate utilized by the myocardium [58], thus precluding us from determining the potential metabolic flexibility of study subjects.

## Conclusions

The current study suggests that an impairment in insulin-stimulated myocardial glucose metabolic rate assessed by ^18^F-FGD PET scan combined with euglycemic hyperinsulinemic clamp is associated with a decrease in myocardial mechano-energetic efficiency, and an increased cardiac workload in subjects with different glucose tolerance status without history of CHD. Myocardial glucose metabolism was the main independent contributor of myocardial MEEi. Overall, these data suggest a predominant role of cardiac insulin resistance in determining alterations of left ventricular mechano-energetic performance, which could at least in part explain its association with CV morbidity.

## Data Availability

The datasets used and analysed during the current study are available from the corresponding author on reasonable request.

## Abbreviations

T2DM: Type 2 diabetes mellitus
CVD: Cardiovascular disease
CHD: Coronary heart disease
MEE: Mechano-energetic efficiency
LV: Left ventricular
SV: Stroke volume
SW: Stroke work
MVO_2_: Myocardial oxygen consumption
MEEi: Indexed MEE
MrGlu: Myocardial glucose metabolic rate
PET: Positron emission tomography
^18^F-FDG: ^18^F-Fluorodeoxyglucose
BMI: Body mass index
BP: Blood pressure
NGT: Normal glucose tolerance
OGTT: Oral glucose tolerance test
ADA: American Diabetes Association
M_FFM_: Insulin-stimulated glucose disposal corrected for fat-free mass
FFM: Fat-free mass
PCARD: Tool specific for cardiac images analysis
VOI: Volume of interest
LVEDV: LV end-diastolic volume
LVESV: LV end-systolic volume
BSA: Body surface area
IVS: Interventricular septum thickness
PWT: Posterior wall thickness
LVM: LV mass
LVMI: LV mass normalized by BSA
SBP: Systolic blood pressure
DP: Double product
HR: Heart rate
FFA: Free fatty acid

## References

[1] Haffner SM, Lehto S, Rönnemaa T, Pyörälä K, Laakso M (1998). Mortality from coronary heart disease in subjects with type 2 diabetes and in nondiabetic subjects with and without prior myocardial infarction. N Engl J Med.

[2] Lee WL, Cheung AM, Cape D, Zinman B (2000). Impact of diabetes on coronary artery disease in women and men: a meta-analysis of prospective studies. Diabetes Care.

[3] Beckman JA, Creager MA, Libby P (2002). Diabetes and atherosclerosis: epidemiology, pathophysiology, and management. JAMA.

[4] Mazzone T, Chait A, Plutzky J (2008). Cardiovascular disease risk in type 2 diabetes mellitus: insights from mechanistic studies. Lancet.

[5] Huang Y, Cai X, Mai W, Li M (2016). Hu Y Association between prediabetes and risk of cardiovascular disease and all cause mortality: systematic review and meta-analysis. BMJ.

[6] Ford ES, Zhao G, Li C (2010). Pre-diabetes and the risk for cardiovascular disease: a systematic review of the evidence. J Am Coll Cardiol.

[7] Huang Y, Cai X, Chen P, Mai W, Tang H, Huang Y, Hu Y (2014). Associations of prediabetes with all-cause and cardiovascular mortality: a meta-analysis. Ann Med.

[8] Tominaga M, Eguchi H, Manaka H, Igarashi K, Kato T, Sekikawa A (1999). Impaired glucose tolerance is a risk factor for cardiovascular disease, but not impaired fasting glucose. The Funagata Diabetes Study. Diabetes Care.

[9] Fiorentino TV, Marini MA, Succurro E, Andreozzi F, Perticone M, Hribal ML, Sciacqua A, Perticone F, Sesti G (2018). One-hour post-load hyperglycemia: implications for prediction and prevention of type 2 diabetes. J Clin Endocrinol Metab.

[10] Marini MA, Succurro E, Frontoni S, Mastroianni S, Arturi F, Sciacqua A, Lauro R, Hribal ML, Perticone F, Sesti G (2012). Insulin sensitivity, β-cell function, and incretin effect in individuals with elevated 1-h postload plasma glucose levels. Diabetes Care.

[11] Bonora E, Kiechl S, Willeit J, Oberhollenzer F, Egger G, Meigs JB, Bonadonna RC, Muggeo M (2007). Insulin resistance as estimated by homeostasis model assessment predicts incident symptomatic cardiovascular disease in Caucasian subjects from the general population: the Bruneck study. Diabetes Care.

[12] Hedblad B, Nilsson P, Engström G, Berglund G, Janzon L (2002). Insulin resistance in non-diabetic subjects is associated with increased incidence of myocardial infarction and death. Diabet Med.

[13] Ingelsson E, Sundström J, Arnlöv J, Zethelius B, Lind L (2005). Insulin resistance and risk of congestive heart failure. JAMA.

[14] Iozzo P, Chareonthaitawee P, Dutka D, Betteridge DJ, Ferrannini E, Camici PG (2002). Independent association of type 2 diabetes and coronary artery disease with myocardial insulin resistance. Diabetes.

[15] Succurro E, Pedace E, Andreozzi F, Papa A, Vizza P, Fiorentino TV, Perticone F, Veltri P, Cascini GL, Sesti G (2020). Reduction in global myocardial glucose metabolism in subjects with 1-hour postload hyperglycemia and impaired glucose tolerance. Diabetes Care.

[16] Ohtake T, Yokoyama I, Watanabe T, Momose T, Serezawa T, Nishikawa J, Sasaki Y (1995). Myocardial glucose metabolism in noninsulin-dependent diabetes mellitus patients evaluated by FDG-PET. J Nucl Med.

[17] Hu L, Qiu C, Wang X, Shao X, Wang Y (2018). The association between diabetes mellitus and reduction in myocardial glucose uptake: a population-based 18F-FDG PET/CT study. BMC Cardiovasc Disord.

[18] Nielsen R, Jorsal A, Iversen P, Tolbod L, Bouchelouche K, Sørensen J, Harms HJ, Flyvbjerg A, Bøtker HE, Wiggers H (2018). Heart failure patients with prediabetes and newly diagnosed diabetes display abnormalities in myocardial metabolism. J Nucl Cardiol.

[19] Succurro E, Vizza P, Papa A, Cicone F, Monea G, Tradigo G, Fiorentino TV, Perticone M, Guzzi PH, Sciacqua A, Andreozzi F, Veltri P, Cascini GL, Sesti G (2022). Metabolic syndrome is associated with impaired insulin-stimulated myocardial glucose metabolic rate in individuals with type 2 diabetes: a cardiac dynamic ^18^ F-FDG-PET Study. Front Cardiovasc Med.

[20] Kofoed KF, Carstensen S, Hove JD, Freiberg J, Bangsgaard R, Holm S, Rabøl A, Hesse B, Arendrup H, Kelbaek H (2002). Low whole-body insulin sensitivity in patients with ischaemic heart disease is associated with impaired myocardial glucose uptake predictive of poor outcome after revascularisation. Eur J Nucl Med Mol Imaging.

[21] Tang K, Lin J, Ji X, Lin T, Sun D, Zheng X, Wang L (2021). Non-alcoholic fatty liver disease with reduced myocardial FDG uptake is associated with coronary atherosclerosis. J Nucl Cardiol.

[22] Losi MA, Wang W, Roman MJ, Lee ET, Howard BV, Devereux RB, de Simone G (2019). Depressed myocardial energetic efficiency increases risk of incident heart failure: the Strong Heart Study. J Clin Med.

[23] de Simone G, Izzo R, Losi MA, Stabile E, Rozza F, Canciello G, Mancusi C, Trimarco V, De Luca N, Trimarco B (2016). Depressed myocardial energetic efficiency is associated with increased cardiovascular risk in hypertensive left ventricular hypertrophy. J Hypertens.

[24] Braunwald E (1971). Control of myocardial oxygen consumption: physiologic and clinical considerations. Am J Cardiol.

[25] Suga H (1990). Ventricular energetics. Physiol Rev.

[26] Bing RJ, Hammond MM, Handelsman JC, Powers SR, Spencer FC, Eckenhoff JE, Goodale WT, Hafkenschiel JH, Kety SS (1949). The measurement of coronary blood flow, oxygen consumption, and efficiency of the left ventricle in man. Am Heart J.

[27] Knaapen P, Germans T, Knuuti J, Paulus WJ, Dijkmans PA, Allaart CP (2007). Myocardial energetics and efficiency: current status of the non- invasive approach. Circulation.

[28] Ingwall JS, Weiss RG (2004). Is the failing heart energy starved? On using chemical energy to support cardiac function. Circ Res.

[29] de Simone G, Chinali M, Galderisi M, Benincasa M, Girfoglio D, Botta I, D’Addeo G, de Divitiis O (2009). Myocardial mechano-energetic efficiency in hypertensive adults. J Hypertens.

[30] Peterson LR, Herrero P, Schechtman KB, Racette SB, Waggoner AD, Kisrieva-Ware Z, Dence C, Klein S, Marsala J, Meyer T, Gropler RJ (2004). Effect of obesity and insulin resistance on myocardial substrate metabolism and efficiency in young women. Circulation.

[31] Mancusi C, de Simone G, Best LG, Wang W, Zhang Y, Roman MJ, Lee ET, Howard BV, Devereux RB (2019). Myocardial mechano-energetic efficiency and insulin resistance in non-diabetic members of the Strong Heart Study cohort. Cardiovasc Diabetol.

[32] Fiorentino TV, Miceli S, Succurro E, Sciacqua A, Andreozzi F, Sesti G (2021). Nonalcoholic fatty liver disease is associated with a decreased myocardial mechano-energetic efficiency. J Intern Med.

[33] Fiorentino TV, Miceli S, Succurro E, Sciacqua A, Andreozzi F, Sesti G (2021). Depressed myocardial mechano-energetic efficiency in subjects with dysglycemia. Diabetes Res Clin Pract.

[34] Succurro E, Miceli S, Fiorentino TV, Sciacqua A, Perticone M, Andreozzi F, Sesti G (2021). Sex-specific differences in left ventricular mass and myocardial energetic efficiency in non-diabetic, pre-diabetic and newly diagnosed type 2 diabetic subjects. Cardiovasc Diabetol.

[35] Succurro E, Marini MA, Fiorentino TV, Perticone M, Sciacqua A, Andreozzi F, Sesti G (2022). Sex-specific differences in prevalence of nonalcoholic fatty liver disease in subjects with prediabetes and type 2 diabetes. Diabetes Res Clin Pract.

[36] American Diabetes Association (2022). Standards of medical care in diabetes-2022. Diabetes Care.

[37] Succurro E, Vizza P, Papa A, Miceli S, Cicone F, Fiorentino TV, Sciacqua A, Andreozzi F, Veltri P, Cascini GL, Sesti G (2022). Effects of 26 weeks of treatment with empagliflozin versus glimepiride on the myocardial glucose metabolic rate in patients with type 2 diabetes: the randomized, open-label, crossover, active-comparator FIORE trial. Diabetes Obes Metab.

[38] DeFronzo RA, Tobin JD, Andres R (1979). Glucose clamp technique: a method for quantifying insulin secretion and resistance. Am J Physiol.

[39] Carson RE (2005). Tracer kinetic modeling in PET. In positron emission tomography.

[40] Vizza P, Guzzi PH, Veltri P, Papa A, Cascini GL, Sesti G, Succurro E. Experiences on quantitative cardiac pet analysis. In: 2016 IEEE international conference on bioinformatics and biomedicine (BIBM). Shenzen, China; 2016. p. 1148–53.

[41] Lang RM, Badano LP, Mor-Avi V, Afilalo J, Armstrong A, Ernande L, Flachskampf FA, Foster E, Goldstein SA, Kuznetsova T, Lancellotti P, Muraru D, Picard MH, Rietzschel ER, Rudski L, Spencer KT, Tsang W, Voigt JU (2015). Recommendations for cardiac chamber quantification by echocardiography in adults: an update from the American Society of Echocardiography and the European Association of Cardiovascular Imaging. J Am Soc Echocardiogr.

[42] Devereux RB, Alonso DR, Lutas EM, Gottlieb GJ, Campo E, Sachs I, Reichek N (1986). Echocardiographic assessment of left ventricular hypertrophy: comparison to necropsy findings. Am J Cardiol.

[43] de Simone G, Kizer JR, Chinali M, Roman MJ, Bella JN, Best LG, Lee ET, Devereux RB, Strong Heart Study Investigators (2005). Normalization for body size and population-attributable risk of left ventricular hypertrophy: the Strong Heart Study. Am J Hypertens.

[44] Gobel FL, Nordstrom LA, Nelson RR, Jorgensen CR, Wang Y (1978). The rate-pressure product as an index of myocardial oxygen consumption during exercise in patients with angina pectoris. Circulation.

[45] Nelson RR, Gobel FL, Jorgensen CR, Wang K, Taylor HL (1974). Hemodynamic predictors of myocardial oxygen consumption during static and dynamic exercise. Circulation.

[46] Salvetti M, Paini A, Bertacchini F, Aggiusti C, Stassaldi D, Capellini S, Arnoldi C, Rizzoni D, Agabiti Rosei C, De Ciuceis C, Muiesan ML (2021). Myocardial mechano-energetic efficiency in primary aldosteronism. J Hypertens.

[47] Cesari M, Frigo AC, Piano S, Angeli P (2021). Low myocardial mechano-energetic efficiency is an independent predictor of prognosis in advanced chronic liver disease. Eur J Gastroenterol Hepatol.

[48] Hinkle PC (2005). P/O ratios of mitochondrial oxidative phosphorylation. Biochim Biophys Acta.

[49] Lionetti V, Stanley WC, Recchia FA (2011). Modulating fatty acid oxidation in heart failure. Cardiovasc Res.

[50] Cook SA, Varela-Carver A, Mongillo M, Kleinert C, Khan MT, Leccisotti L, Strickland N, Matsui T, Das S, Rosenzweig A, Punjabi P, Camici PG (2010). Abnormal myocardial insulin signalling in type 2 diabetes and left-ventricular dysfunction. Eur Heart J.

[51] Tan Y, Zhang Z, Zheng C, Wintergerst KA, Keller BB, Cai L (2020). Mechanisms of diabetic cardiomyopathy and potential therapeutic strategies: preclinical and clinical evidence. Nat Rev Cardiol.

[52] Sesti G (2006). Pathophysiology of insulin resistance. Best Pract Res Clin Endocrinol Metab.

[53] Juszczyk A, Jankowska K, Zawiślak B, Surdacki A, Chyrchel B (2020). Depressed cardiac mechanical energetic efficiency: a contributor to cardiovascular risk in common metabolic diseases-from mechanisms to clinical applications. J Clin Med.

[54] Tran P, Maddock H, Banerjee P (2022). Myocardial fatigue: a mechano-energetic concept in heart failure. Curr Cardiol Rep.

[55] Gerber BL, Ordoubadi FF, Wijns W, Vanoverschelde JL, Knuuti MJ, Janier M, Melon P, Blanksma PK, Bol A, Bax JJ, Melin JA, Camici PG (2001). Positron emission tomography using (18)F-fluoro-deoxyglucose and euglycaemic hyperinsulinaemic glucose clamp: optimal criteria for the prediction of recovery of post-ischaemic left ventricular dysfunction: results from the European Community Concerted Action Multicenter Study on Use of (18)F-Fluoro-Deoxyglucose Positron Emission Tomography for the Detection of Myocardial Viability. Eur Heart J.

[56] Jenkins DG, Quintana-Ascencio PF (2020). A solution to minimum sample size for regressions. PLoS ONE.

[57] Schisterman EF, Cole SR, Platt RW (2009). Overadjustment bias and unnecessary adjustment in epidemiologic studies. Epidemiology.

[58] Masi S, Lautamäki R, Guiducci L, Di Cecco P, Porciello C, Pardini S, Morales MA, Chubuchny V, Salvadori PA, Emdin M, Sironi AM, Knuuti J, Neglia D, Nuutila P, Ferrannini E, Iozzo P (2012). Similar patterns of myocardial metabolism and perfusion in patients with type 2 diabetes and heart disease of ischaemic and non-ischaemic origin. Diabetologia.

